# Oxidative Stress in Radiation-Induced Cardiotoxicity

**DOI:** 10.1155/2020/3579143

**Published:** 2020-03-01

**Authors:** Zhang Ping, Yang Peng, Hong Lang, Cai Xinyong, Zeng Zhiyi, Wu Xiaocheng, Zeng Hong, Shao Liang

**Affiliations:** ^1^Department of Neurology, Jiangxi Provincial People's Hospital Affiliated to Nanchang University, Nanchang, 330006 Jiangxi, China; ^2^Department of Cardiology, Jiangxi Provincial People's Hospital Affiliated to Nanchang University, Nanchang, 330006 Jiangxi, China

## Abstract

There is a distinct increase in the risk of heart disease in people exposed to ionizing radiation (IR). Radiation-induced heart disease (RIHD) is one of the adverse side effects when people are exposed to ionizing radiation. IR may come from various forms, such as diagnostic imaging, radiotherapy for cancer treatment, nuclear disasters, and accidents. However, RIHD was mainly observed after radiotherapy for chest malignant tumors, especially left breast cancer. Radiation therapy (RT) has become one of the main ways to treat all kinds of cancer, which is used to reduce the recurrence of cancer and improve the survival rate of patients. The potential cause of radiation-induced cardiotoxicity is unclear, but it may be relevant to oxidative stress. Oxidative stress, an accumulation of reactive oxygen species (ROS), disrupts intracellular homeostasis through chemical modification and damages proteins, lipids, and DNA; therefore, it results in a series of related pathophysiological changes. The purpose of this review was to summarise the studies of oxidative stress in radiotherapy-induced cardiotoxicity and provide prevention and treatment methods to reduce cardiac damage.

## 1. Introduction

Radiotherapy plays an important role in the treatment of many cancers. As the use of radiotherapy is becoming increasingly frequent, and since the overall patient survival rate is high, the risks associated with radiotherapy must be carefully considered. Among these risks, cardiovascular diseases (CVDs) have always attracted much attention, since CVD is the leading cause of nonmalignant tumor-related deaths in cancer survivors [1].

In a clinical setting, gamma rays and X-rays are the most commonly used types of ionizing radiation. Radiation-induced cardiotoxicity depends on the type and dose of radiation [2]. Clinical studies have shown that a radiation dose of 1–4 Gy promotes the development of CVD and inflammation [3]. A radiation dose of 5–8 Gy increases the possibility of myocardial infarction (MI), angina, pericarditis, and decreased left ventricular diameter, while radiation doses of more than 8 Gy cause myocardial fibrosis, which usually occurs after irradiation for Hodgkin's lymphoma (HL) [4–6]. At doses above 30 Gy, the risk of radiation-related heart disease becomes significant if the patient is exposed for a year or two; however, the latent period of radiation-related heart disease is longer and disease can occur more than a few decades later if exposure has been at lower radiation doses [7]. Studies have shown that in an experiment with a larger-than-average cardiac radiation dose, the risk of heart death increased significantly by approximately 3% per Gy of radiation dose [2, 7]. Radiotherapy is now used for approximately half of all malignant tumors and is the basic treatment for HL and breast cancer [8]. Nevertheless, radiation-induced coronary heart disease is the second most common cause of mortality and incidence in patients with breast cancer and HL treated with radiotherapy [9]. The use of high doses of radiation in the treatment of cancer has been shown to damage heart tissue, leading to cardiac dysfunction and CVD [2]. The available data show that the higher the radiation dose, the stronger is the cardiotoxicity, and the risk of cardiovascular complications is also increased. Moreover, the risk of cardiovascular complications in patients who received radiotherapy for left breast cancer was significantly higher than the risk in patients who received radiotherapy for right breast cancer [10]. Although the benefit of radiotherapy is obvious, increasing attention has been paid to the cardiac damage induced by radiotherapy, which would suggest limiting the dose and use of radiotherapy in cancer patients [11]. Modern radiotherapy techniques may not have decreased cardiac toxicity even though they have reduced the exposure of the heart to radiation [12]. A number of studies have emphasized the role of oxidative stress and inflammation in radiation-induced cardiovascular damage and have shown that most chemotherapeutic drugs and radiotherapy can increase oxidative stress. Therefore, antioxidative stress has become an important therapeutic target for radiation-induced cardiotoxicity.

Chest radiotherapy is used to effectively treat some malignant tumors, such as HL and breast cancer. However, the incidence of cardiovascular events in these patients has increased for years, especially among young survivors who do not have traditional risk factors [13]. Oxidative stress in cells is the main factor of CVD [14]. Oxidative stress represents the imbalance between the production of reactive ROS and the scavenging of ROS by the cell antioxidant defense system, thus, mediating the damage of cell structure, including lipids, proteins, and DNA [15]. Oxidative stress and ROS production have always been considered to be important pathophysiological mediators leading to CVD. Chronic and acute overproduction of ROS under pathophysiological conditions is an important part of the development of CVD [16]. In general, there is a considerable amount of data indicating that oxidative stress and ROS are related to the pathophysiology of CVD [17]. The effect of oxidative stress on radiation-induced cardiotoxicity may be mainly owing to the oxidative damage of biological macromolecules (DNA, protein, and lipids) and a series of molecular signaling pathways mediated by ROS through the excessive production of ROS.

### 1.1. Formation of ROS

**Figure 1:**
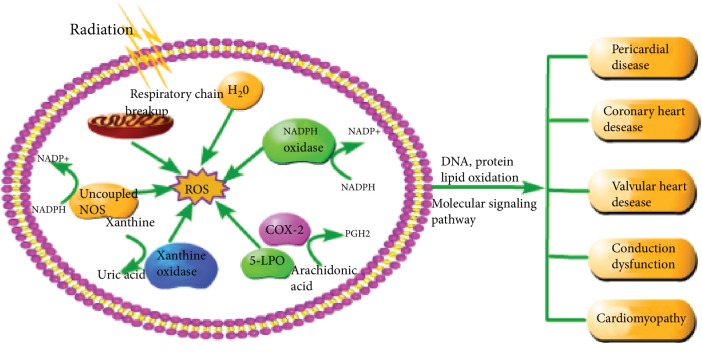
Formation of ROS after radiation and the general manifestation of cardiotoxicity. As described in the article, the sources of ROS are varied, even under the radiation conditions. IR can directly cause the respiratory chain of mitochondria to breakup and cause the decomposition of water molecules, leading to respiratory chain dysfunction and ROS production, reducing antioxidant capacity. NADPH oxidase is a family of multisubunit complex enzymes that catalyze the conversion of oxygen into O_2_^−^ by using NADPH as an electron source, which is present in vascular endothelia cells, smooth muscle cells, fibroblasts, and cardiomyocytes. The mechanism of uncoupled NOSs is similar to NADPH oxidase. Xanthine oxidase catalyzes the conversion of xanthine to uric acid while H_2_O_2_ and O_2_^−^ are generated at the same time. In addition, COX-2 and 5-LPO produce PGH2 accompanied by ROS formation during catalytic arachidonic acid metabolism. ROS can interact with macrobiomolecules (DNA, protein, and lipid), causing oxidation of DNA, proteins, and lipids and cause cardiac damage through some signaling pathways, which are described in article above. The cardiac damage includes pericardial disease, coronary heart disease, heart valve disease, conduction disorders, and cardiomyopathy.

As illustrated in Figure 1, oxidative stress refers to the imbalance between oxidants and antioxidants, which is favorable to oxidants and leads to harmful effects [15]. Oxidants, also known as ROS, include superoxide radical anions (O_2_^−^), hydrogen peroxide (H_2_O_2_), hydroxyl radical (OH^−^), and singlet oxygen. In addition, they also contain some nitrogen oxides, lipid peroxide free radical (LOO), and hypochlorous acid (HOCl) [14, 18]. ROS in the cardiovascular system come from endogenous sources, such as NADPH oxidases, mitochondria, xanthine oxidases, cyclooxygenase (COX), lipoxygenase (LPO), and uncoupled nitric oxide synthases (NOSs); exogenous sources of ROS include chemical toxins, radiation, ultraviolet rays, cigarette smoke, and drugs. These sources result in elevated ROS concentrations and subsequent cardiovascular tissue damage [14, 19]. Mitochondria are the major sites of intracellular oxygen consumption, and the respiratory chain of mitochondria is the main source of ROS, which has an important effect on the cardiovascular system [20]. IR can directly cause the respiratory chain of mitochondria to breakup, leading to respiratory chain dysfunction and thus reducing ATP production, increasing ROS production, reducing antioxidant capacity, and inducing apoptosis [21]. NADPH oxidase (NOXs) is the main enzymatic source of ROS in the cardiovascular system [22]. Among them, NOX2 and NOX4 are the most abundant in the heart and are expressed in cardiomyocytes and endothelial cells. These enzymes catalyze the transfer of electrons from NADPH to oxygen molecules to produce oxygen free radicals [23]. In addition, the late effects of radiation therapy are caused by the ionization of water molecules in the surrounding environment and the generation of chronic free radicals induced by radiation [24]. Radiation can not only change the upstream source of ROS but also change the balance of endogenous antioxidant defense mechanisms, including glutathione, ascorbic acid, catalase, and superoxide dismutase (SOD) [25]. The inhibition of antioxidant enzymes is one of the important effects of ionizing radiation on irradiated organs and off-site organs, which leads to the production and accumulation of ROS [26]. ROS can react randomly with cellular lipids, proteins, and nucleic acids, and cause oxidative stress, thus damaging these macromolecules [25].

### 1.2. ROS-Mediated Oxidative Damage to Biomacromolecules

ROS are a kind of small reaction molecule and play a key role in regulating cell function and biological process [27]. ROS are highly reactive and can interact with biological macromolecules, such as DNA, proteins, and lipids, and can produce various pathological manifestations via changing the function of these macromolecules [15].

#### 1.2.1. DNA Oxidation

Radiation directly and indirectly affects heart tissue. Radiation may interact directly with DNA and cause DNA damage [28]. The ROS produced by the radiation decomposition of water molecules and the surrounding DNA molecules are considered the indirect effect [29]. The IR-induced DNA damage is mainly caused by indirect effects, including base damage, cross-linking, single-strand break (SSBS), and double-strand break (DSBS). In these lesions, they can be either alone or in combination with another, resulting in complex DNA damage, with DSBS being the most severe [30]. The oxidative damage of DNA can change gene expression, resulting in protein modification, cell death, and genome instability [29]. The mitochondrial respiratory system is the main source of ROS in cells. They are by-products of the transfer of electrons from NADH or FADH to molecular oxygen under normal physiological conditions, and these toxic by-products are treated by antioxidants and free radical scavenging enzymes, including manganese superoxide dismutase (Mn-SOD), catalase (CAT), and glutathione peroxidase (GPX) in mitochondria [31]. However, owing to the lack of chromatin structure, histone protection, and an inefficient repair system, mtDNA is vulnerable to oxidative stress-related damage. If not repaired, it will lead to the destruction of the electronic transport chain and produce more ROS, which exceeds the protective ability of the antioxidant system [20, 31]. Reactive oxygen-induced mitochondrial DNA oxidative damage and mitochondrial damage lead to the pathological production of ROS, and the formation of the vicious cycle leads to cell energy consumption and apoptosis. The accumulation of mitochondrial DNA oxidative damage can lead to mitochondrial dysfunction, which is an important cause of some human diseases, including CVD [20].

#### 1.2.2. Protein Oxidation

Oxidative stress by ROS produced after radiation can lead to chain breaks, protein charge changes, protein cross-linking, and oxidation of specific amino acids, resulting in increased susceptibility to a particular protease-degrading protein. Cysteine and methionine residues in the protein are particularly sensitive to oxidation. The oxidation of the base or methionine residue can cause conformational change, protein expansion, and degradation [15]. Oxidative stress-induced protein oxidative modification before protein degradation or inactivation increased. A previous study has shown that homocysteamine was common in irradiated endothelial cells, and the increased level of homocysteine was associated with various human CVDs, including atherosclerosis [32]. Oxidative damage of proteins in vivo may affect the function of receptors, enzymes, transporters, etc., and cause secondary damage to other biomolecules [33]. As one of the main protein targets of ROS, Ca^2+^/calmodulin-dependent protein kinase II (CaMKII) plays a key role in the pathophysiology of CVDs [34]. CaMKII is a serine-threonine kinase. There are four known subtypes: *α*, *β*, *γ*, and *δ*. *δ* subtypes play a dominant role in the heart. CaMKII is activated not only by binding calcium-bound calmodulin (Ca^2+^/CaM) but also by oxidation [35]. The oxidation of CaMKII is carried out by ROS, and in the presence of ROS, methionine 281 and 282 in CaMKII are oxidized [36]. In the domain of Ca^2+^/CaM-binding CaMKII, oxidation of M281/282 leads to enzyme activation which inhibits the recombination between a regulatory subunit and a catalytic subunit, thus perpetuating the activity of CaMKII [34]. The effect of CaMKII enhanced by oxidation is complex and widely present in the cardiac muscle cells, and the excessive activation of CaMKII may be related to cardiomyopathy and abnormal excitation-contraction coupling (ECC), heart failure, and arrhythmia [35, 36].

#### 1.2.3. Lipid Peroxidation

The main mechanisms for free radical-induced cell and tissue damage include the formation of lipid peroxides in the cell membrane and organelles. This process begins when the free radical is extracted from polyunsaturated fatty acids (PUFA) to form a fatty acid radical. The biologically active lipid peroxidation radical can react with other lipids, proteins, or nucleic acids to facilitate the transfer of electrons and the oxidation of the substrate. These organic radicals perpetuate the chain reaction by attacking additional side chains, resulting in the formation of a lipid peroxide [37]. In general, lipid peroxide can be described as the process of oxidants (such as free radicals) attacking lipids containing carbon double bonds, especially PUFA [28]. PUFA, in particular linoleic acid and arachidonic acid, are an important target of lipid peroxide. Malondialdehyde (MDA) and 4-hydroxy-2-non-ene (HNE) are the most important products of lipid oxidation [21]. ROS can induce lipid peroxide and destroy the bilayer arrangement of membrane lipids, which may lead to the inactivation of membrane-binding receptors and enzymes and increase the permeability of tissues. Lipid peroxidation products, such as MDA and unsaturated aldehyde, can inactivate many cell proteins by forming protein cross-linking. HNE can lead to intracellular glutathione (GSH) depletion and induce peroxide production, activate epidermis growth factor receptor, and induce fibronectin production [15]. Because the myocardial cell membrane is rich in phospholipids which are particularly sensitive to oxidative stress and the antioxidant capacity is low, the myocardium is particularly vulnerable to oxidative damage of free radicals produced by ionizing radiation. Lipid peroxidation in the myocardial cell membrane can lead to injury of the myocardial structure and impaired function [38]. In addition, lipid peroxide also exists in the oxidation of low-density lipoprotein (LDL) [28]. Atherosclerosis is the pathological basis of coronary heart disease and is closely related to oxidative LDL. Infiltration and accumulation of low-density lipoprotein cholesterol (LDL) in the endothelial cells occur after endothelial injury. Monocytes differentiate into macrophages and express scavenging receptors (SRS), such as CD36, SRA, and LOX-1 [27]. Oxidized LDL (OxLDL) attracts macrophages directly and is phagocytic by macrophages. Because OxLDL is resistant to macrophage lysosomal acidic proteolytes, the OxLDL collected by macrophages is not digested and decomposed by macrophages. Over time, OxLDL accumulates more and more in macrophages and converts macrophages into cells containing a lot of fat in cytoplasm, called foam cells [19]. In addition, ROS induces the expression of SRS in smooth muscle cells and converts them into foam cells. The presence of foam cells in the arterial wall is a sign of early atherosclerosis. Various studies have shown that OxLDL can induce endothelial cells, vascular smooth muscle cells, and macrophages to produce ROS, and ROS and cytokines released by inflammatory cells may stimulate smooth muscle cell migration and collagen synthesis, leading to the formation of atherosclerotic plaques [27].

### 1.3. Molecular Signaling Pathway of Cardiac Toxicity Mediated by ROS

Irradiation of normal tissues can lead to a sharp increase in ROS and reactive nitrogen species (RNS), which include nitric oxide (NO), nitrogen dioxide (NO_2_), and peroxynitrite (ONOO^−^). ROS/RNS are free radicals which are associated with the oxygen atom (O) or their equivalents and have stronger reactivity with other molecules. ROS/RNS as intracellular and intercellular signals change the function of cells and tissues [39]. In the case of chest irradiation, exposure of the heart, blood vessels, and other tissues leads to tissue remodeling and adverse cardiovascular events. This complex process is composed of a large number of interacting molecular signals, including cytokines, chemotactic factors, nuclear transcription factors, and growth factors [28].

#### 1.3.1. TGF-*β*1 and Oxidative Stress

**Figure 2:**
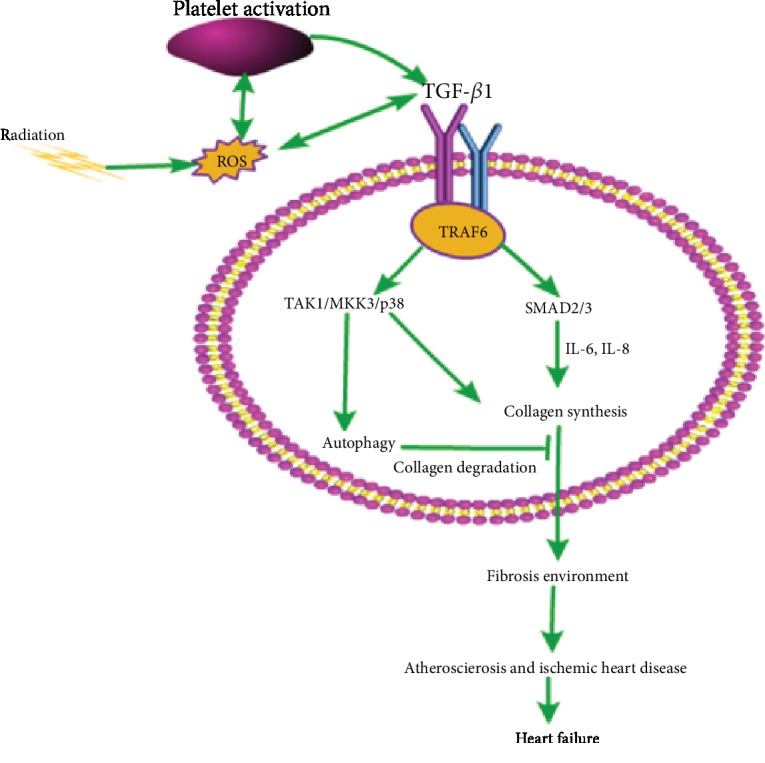
Oxidative stress in TGF-*β*1-mediated fibrosis. Radiation induces reactive oxygen species (ROS), and ROS can activate TGF-*β*1, resulting in the generation of thrombin and the activation of platelets to promote further production of ROS and TGF-*β*1. TGF-*β*1 binds to Type I (TbRI) and Type II (TbRII) transmembrane receptors, activating the Smad pathway and the TAK1/MKK3/p38 pathway through the specific nuclear signaling transduction molecule, TRAF6. The Smad pathway requires a number of inflammatory cytokines, in particular IL-6 and IL-8, to mediate collagen synthesis. TGF-*β*1 activates the TAK1/MKK3/p38 signaling pathway and downregulates autophagy-mediated collagen degradation, which may further promote the synthesis of collagen, form a fibrotic environment, cause atherosclerosis and ischemic heart disease, and may even lead to heart failure.

As shown in Figure 2, radiation-induced vascular injury and endothelial dysfunction are partly mediated by transforming growth factor- (TGF-) *β*, which plays a key role in radiation-induced fibrosis [40]. ROS produced by radiation is an immediate activating agent of TGF-*β*1, and the activated TGF-*β*1 initiates an upregulation of collagen synthesis in a dose-dependent manner [41]. ROS can result in TGF-*β*1 activation, thrombin production, platelet activation, and proinflammatory signal activation to promote myofibroblast accumulation and extracellular matrix (ECM) production. ROS is activated through a forward feedback loop by TGF-*β*1 to amplify these fibrosis signals. Radiation-induced ROS release TGF-*β*1 together with ECM, causing oxidative damage to DNA, proteins, and lipids [42]. TGF-*β*1 activates two signal pathways, the Smad pathway and the TAK1/MKK3/p38 pathway, through a special nuclear signal transducer molecule called TRAF6. Collagen synthesis is controlled by these two pathways in the steady state [42, 43]. In the classical signaling pathway, TGF-*β* recruits a complex of Type I (TbRI) and Type II (TbRII) transmembrane receptors to induce the phosphorylation of Smad protein and induce the accumulation of Smad protein in the nucleus to regulate the expression of target genes [44]. Many inflammatory cytokines, especially IL-6 and IL-8, are involved in the Smad pathway, which mediate collagen synthesis and lead to rational fibrosis and dysfunction in heart disease. TAK1/MKK3/p38 signal transduction is the key to autophagy-dependent collagen degradation. TGF-*β*1 can also reduce the synthesis of proteolytic enzymes and increase the synthesis of protease inhibitors to degrade matrix and downregulate autophagy-mediated collagen degradation, which may further promote TGF-*β*1-related events [42]. Radiation-induced coronary heart disease (RICHD) is a late effect of radiation exposure, with tissue fibrosis as the common end point. Fibrosis of the coronary and carotid arteries may destroy the normal blood supply of the heart muscle, leading to ischemia and heart failure [30].

#### 1.3.2. IGF-1 and Oxidative Stress

**Figure 3:**
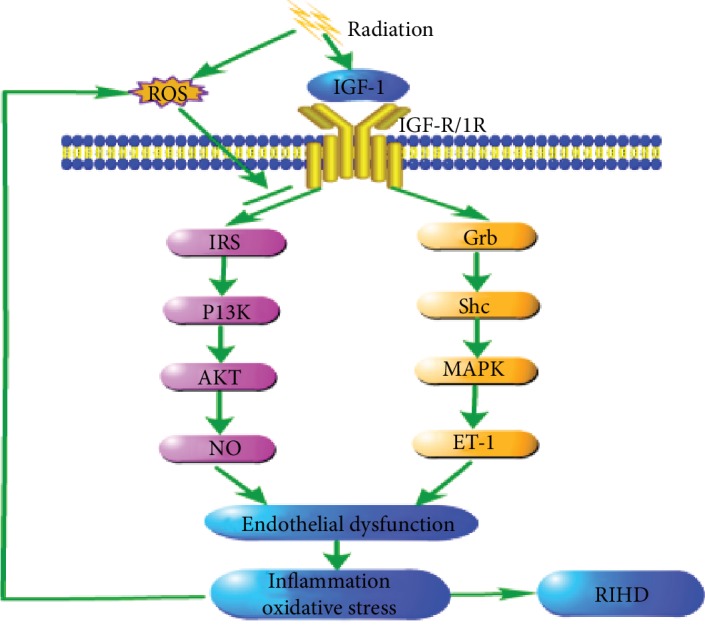
Radiation induced IGF-1 selective resistance. Insulin-like growth factor-1 (IGF-1) is structurally similar to insulin. It can activate the IRS/PI3K/Akt pathway (PI3K dependent) and the Grb/Shc/MAPK pathway (PI3K independent) through IGF-R, IR, or the hybrid receptor. However, radiation can inhibit the anti-inflammatory and antioxidant stress of the IRS/PI3K/Akt signaling pathway but not inhibit the inflammatory MAPK kinase pathway, which leads to the transformation of the IGF-1 signal to the oxidation and inflammatory environment, which leads to selective resistance of IGF-1. ROS can also cause selective inhibition of IRS/PI3K/Akt pathway and even enhance the MAPK signaling pathway. IGF-1 selective resistance causes an imbalance between NO and ET-1 production, resulting in endothelial dysfunction, resulting in oxidative stress and inflammatory level and further leads to the occurrence and development of radiation-induced heart diseases.

Insulin-like growth factor-1 (IGF-1) is a primary mediator of the effects of growth hormones and is itself structurally similar to insulin [45]. The assembly structure of the IGF-1 receptor (IGF-1R) is the same as that of the insulin receptor, and the homology of kinase domain *β* is more than 80%. Therefore, IGF-1 and insulin have at least two main signal transduction pathways (PI3K-dependent and PI3K-independent pathways) through their homologous receptor and cross-activation [46]. Among them, the most important cardiovascular function of IGF-1 comprises the two main ways of regulating vascular tension: through the anti-inflammatory/antioxidant stress IRS/PI3K/Akt pathway and through the proinflammatory and oxidative stress Grb/Shc/MAPK (PI3K-independent) pathway [47]. The activation of the IRS/PI3K/Akt pathway leads to eNOS phosphorylation of Ser1179 and the production of nitric oxide (NO) within minutes. In the presence of BH4, eNOS produces NO during the conversion of L-arginine to L-citrulline. In the absence of BH4, eNOS uncoupling catalysis forms a superoxide instead of NO [17]. NO, a powerful vasodilator with antithrombotic and anti-inflammatory characteristics, is involved in oxidative stress and chronic endothelial activation and inflammation [48]. NO has an important protective effect on maintaining normal cardiovascular function. Excessive ROS production can reduce the bioavailability of NO, which is the main factor for the occurrence and development of CVDs [49]. For example, the activation of NF-*κ*B after radiation is inhibited by NO, and therefore, a decrease in the bioavailability of NO may promote the development of vascular inflammation and atherosclerosis [40]. IGF-1 stimulates the expression of endothelin-1 (ET-1) by activating the Shc/Grb/MAPK pathway via IGF-R, IR, or a hybrid receptor [17]. ET-1 is a powerful vasoconstrictive peptide secreted by endothelial cells. It can antagonize the vasodilation effect of NO and has the characteristics of promoting oxidation and inflammation [50]. Research on mice has shown that IGF-1 provides radiation protection, and the use of IGF-1 inhibitors enhances the sensitivity of cancer cells to radiotherapy [51]. As demonstrated by Kenchegowda et al. in 2018, IGF-1 levels were increased in irradiated minipigs but a positive correlation was observed between elevated IGF-1 levels after radiation and cardiac adverse events. It was further proven that the IGF-1/PI3K/Akt pathway was inhibited while the MAPK pathway remained active, resulting in the transformation of the IGF-1 signal to an oxidative and proinflammatory environment, which led to selective resistance of IGF-1 [45]. Furthermore, other pathological stimuli such as ROS and inflammatory cytokines can cause selective inhibition of the IRS/PI3K/Akt pathway and even enhance the MAPK signal pathway [47]. Selective IGF-1 resistance has recently been considered to be a new radiation injury mechanism related to inflammation and endothelial dysfunction [52]. The inhibition of the IRS/PI3K/Akt/eNOS signal transduction pathway and the activation of the Grb/Shc/MAPK/ET pathway lead to an imbalance between NO and ET-1, resulting in endothelial dysfunction and CVD [47]. Endothelial dysfunction refers to a complex pathological state, which is characterized by a series of events, including damage of the endothelial barrier, damage of vasodilation and contraction, decrease of NO production, increase of adhesion molecule expression, increase of cytokine levels, and ROS produced by endothelial cells. Endothelial dysfunction helps promote fibrosis and an inflammatory environment, leading to fibrosis as a secondary effect, which is a common feature of radiation-induced tissue damage [32]. The injury of vascular endothelial cells plays an important role in radiation-induced heart injury [48]. The overall results of IGF-1 resistance include vasoconstriction, decrease of blood flow, hypoperfusion, and a further increase of oxidative stress, which leads to the increase of ROS level and the formation of a vicious cycle. ROS can promote the inhibition of the PI3/Akt pathway, decrease the bioavailability of NO, and induce the production of ET-1, leading to vascular dysfunction, inflammation, and thrombosis, and ultimately to the occurrence and development of atherosclerosis [45]. Moreover, endothelial injury is often accompanied by myocardial injury, which results in collagen deposition in the capillary cavity, stenosis of blood vessels, and deterioration of myocardial blood supply, forming a vicious cycle of myocardial blood supply and continuous fibrosis and remodeling [53]. This process is described in Figure 3.

#### 1.3.3. NF-*κ*B and Oxidative Stress

**Figure 4:**
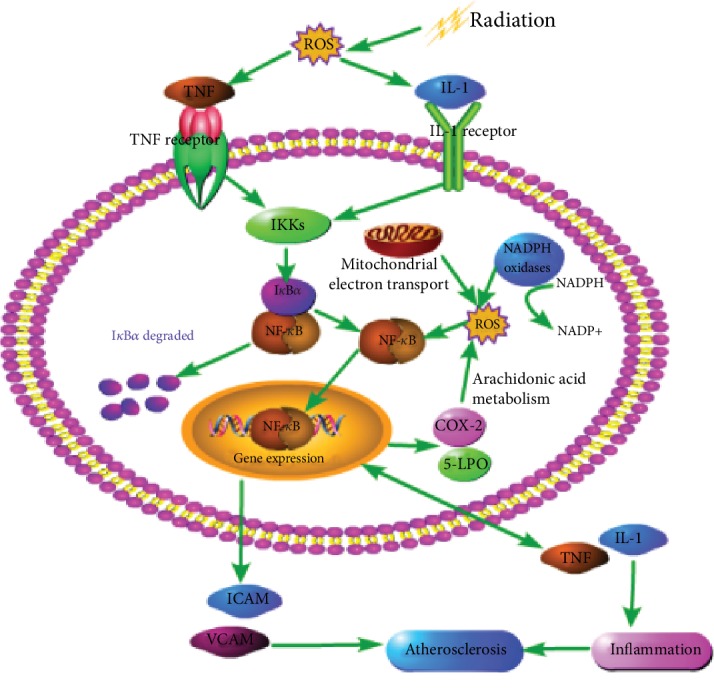
The relationship between oxidative stress and NF-*κ*B. In normal cases, NF-*κ*B combines with the inhibitor protein (IKB) to form a protein complex and remain in an inactive state. However, when the cells are exposed to ionizing radiation, oxidative stress reactions, such as NADPH oxidase activity, improved and mitochondrial electron transport is impaired and occurs rapidly, and reactive oxygen species is excessively generated in response to oxidative stress, which stimulates the inflammatory cells to produce IL-1 and TNF. IL-1 and TNF bind to the IL-1 receptor and the TNF receptor, respectively, to activate the downstream NF-*κ*B signal. The IKB protein is phosphorylated and NF-*κ*B is activated and translocated to the nucleus, which binds to the various promoter regions of its target gene and induces the transcription of a corresponding inflammation-related gene. The activated NF-*κ*B induced the expression of COX-2 and 5-LPO, resulting in the generation of ROS, the formation of a positive feedback loop, and increased levels of inflammation and oxidative stress, and the intracellular ROS can activate NF-*κ*B directly. In addition, the activation of NF-*κ*B also results in an increase in the expression of intercellular adhesion molecules (ICAM) and vascular cell adhesion molecules (VCAM). In addition, NF-*κ*B upregulated the expression of cytokines such as IL-1 and TNF. These cytokines increase inflammation not only by attracting white blood cells but also by activating NF-*κ*B. Thus, the inflammation state of atherosclerosis may again form a positive self-regulatory loop, forming proinflammatory and fibrotic environments, leading to tissue remodeling, myocardial fibrosis, and atherosclerosis.

As shown in Figure 4, ROS form an important intermediate and second messenger activated by NF-*κ*B through upstream stimuli, such as tumor necrosis factor (TNF) and interleukin-1 (IL-1) [54]. NF-*κ*B belongs to the family of inducible transcription factors. NF-*κ*B regulates different biological processes, including immune response, inflammation, cell proliferation, and apoptosis. NF-*κ*B binds to inhibitor protein IKB (I*κ*B*α*, in particular, has been extensively studied) to form protein complexes and remains inactive [28]. When cells are exposed to ionizing radiation, oxidative stress occurs rapidly. The activation of the TNF receptor and the IL-1 receptor, the upstream receptors of the typical signaling pathway of NF-*κ*B, further activates the IKK complex (primarily IKK*β*) composed of IKK*α* and IKK*β* (catalytic kinase), which phosphorylates I*κ*B*α* on serines 32 and 36, resulting in the phosphorylation and degradation of IKB protein and the activation and translocation of NF-*κ*B to the nucleus [55]. It can bind to various promoter regions of its target genes and induce transcription of corresponding genes, most of which are involved in the regulation of inflammation [28, 56]. NF-*κ*B target genes include COX-2 and 5-LPO, and these two are the sources of ROS generation. 5-LPO, similar to COX-2, produces prostaglandin H2 (PGH2) accompanied by ROS formation during catalytic arachidonic acid metabolism, and intracellular ROS production can directly activate NF-*κ*B [55]. Therefore, a positive feedback loop can be formed between NF-*κ*B and ROS. ROS activates NF-*κ*B, which in turn increases the production of ROS by increasing the expression of COX-2 and 5-LPO [19]. In addition, NF-*κ*B is activated by oxidative stress after radiation, which targets the production of many genes related to inflammation, including intercellular adhesion molecules (ICAM), vascular cell adhesion molecules (VCAM), and cytokines, and promotes the upregulation of thrombus formation markers [40]. Furthermore, NF-*κ*B upregulates the expression of cytokines such as IL-1 and TNF. These cytokines increase inflammation not only by attracting white blood cells but also by activating NF-*κ*B [57, 58]. Thus, the inflammatory state of atherosclerosis may again form a positive self-regulatory loop [19]. This proinflammatory environment, coupled with collagen deposition and increased fibroblasts, leads to tissue remodeling, myocardial fibrosis, and atherosclerosis, which is one of the end-stage symptoms of radiation-induced heart disease [54]. In addition, the inflammation regulated by NF-*κ*B is also associated with myocardial injury induced by radiation. Because of the increased expression of intercell adhesion molecules and vascular cell adhesion molecules, the adhesion of leukocytes to the endothelial cells and the thrombus can prevent the vascular cavity from causing microthrombus and vascular occlusion, thereby causing filling defects and focal ischemia, followed by further myocardial cell death and fibrosis [28].

#### 1.3.4. IL-4 and Oxidative Stress

IL-4 has long been considered to be an effective fibrogenic factor because it can induce the synthesis of ECM protein, collagen, and fibronectin [59]. Recent studies have shown that IL-4 and IL-13 are of importance in the late cardiac effects induced by ionizing radiation [60]. IL-4 and IL-13 are mainly secreted by Th2 lymphocytes and act on a variety of tissues to stimulate collagen synthesis [61]. These cytokines may induce chronic oxidative stress, inflammation, and fibrosis by upregulating dual oxidase 1 (DUOX1) and dual oxidase 2 (DUOX2). DUOX is also known as an NADPH oxidase, one of the main sources of ROS [60]. DUOX1 and DUOX2 are two membrane-dependent subfamilies of oxidoreductase, which catalyze the conversion of oxygen (O_2_) to hydrogen peroxide (H_2_O_2_). The two enzymes are very similar and can produce a large amount of H_2_O_2_ after activation. The abnormal upregulation of these enzymes may lead to the destruction of normal function and the increase of oxidative damage [62]. Chronic oxidative stress and inflammation can lead to fibrosis and hypertrophy, which may undermine normal cardiac function and myocardial blood supply [63]. A study by Farhood et al. in 2017 showed that irradiation increased the level of IL-4 but not the level of IL-13 in rats. The results of real-time PCR showed that radiation induced the upregulation of IL4ra1 (IL-4 receptor), DUOX1, and DUOX2; the changes induced by IL4ra1 and DUOX1 were the most obvious [62]. In addition to DUOX1 and DUOX2, IL-4 can also induce the upregulation of TGF-*β*, a powerful stimulator of fibrosis [63]. As mentioned earlier, the activation of the TGF-*β* pathway mediates collagen synthesis, promotes tissue fibrosis, and plays an important role in late radiation-induced cardiac toxicity.

### 1.4. Clinical Manifestations of Radiation-Induced Cardiotoxicity

The use of high doses of radiation in cancer treatment has been shown to damage heart tissue, leading to cardiac dysfunction and CVD [2]. Radiation-induced CVDs can include pericardial disease, coronary artery disease, valvular heart disease, conduction disease, and cardiomyopathy [8, 13]. Heart disease caused by radiotherapy develops slowly and occurs 20–30 years after radiotherapy [24].

#### 1.4.1. Radiation-Induced Pericardial Disease

Pericardial disease is a common and classic complication of mediastinal radiotherapy; its types include acute pericarditis, pericardial effusion, delayed thickening, and constrictive pericarditis [13]. In a postmortem study, 20 cancer patients with a history of mediastinal radiotherapy were examined. 70% (14) of the patients had pericardial disease, the most common of which was exudative or constrictive disease [64]. The symptoms of these patients developed at different times and appeared three to five years after radiation [13]. Acute pericarditis usually occurs during radiotherapy or within a few weeks after radiotherapy, and the exudate is fibroid. It is usually caused by high-dose (40 Gy) radiation therapy for mediastinal tumors, especially HL [40]. However, acute pericarditis is now rare owing to new radiotherapy techniques, such as deep inspiratory breath-holding (DIBH) and accelerated partial breast irradiation (APBI), which significantly reduce the dose of cardiac radiation exposure [62, 65]. The patients could show fever, pleurisy and chest pain, abnormal electrocardiogram, and slight elevation of cardiac markers. The disease is usually self-limited and effectively treated by nonsteroidal anti-inflammatory drugs (NSAID) and colchicine [62]. Radiation-induced chronic pericarditis often has no relative symptoms, but it can be disguised as angina or it could coexist with angina [9]. It is important to note, however, that chronic pericardial diseases can develop months to years after radiotherapy treatment and may lead to large pericardial fluid accumulation. Excessive accumulation may lead to pericardial tamponade. Symptoms and signs, such as dyspnea, accompanied by distal heart sound, hypotension, and a dilated jugular vein, may be diagnostic indicators. Echocardiography is still the golden standard for deterministic diagnosis [53]. Constrictive pericarditis is usually one of the most serious complications, and diuretics are usually the first line of defense; however, the final treatment is pericardiectomy [8, 13].

#### 1.4.2. Radiation-Induced Coronary Heart Disease

RICHD is the second most common cause of onset and death in patients with breast cancer, HL, and other common mediastinal malignant tumors. The risk of RICHD increases with the increase of the radiation dose. Exposed patients may develop a variety of symptoms after decades of treatment, including asymptomatic myocardial perfusion defects, stomatal processes, triple-vessel disease, and sudden cardiac death [9]. In terms of pathology, RICHD and common coronary heart disease differ in some respects. RICHD lesions tend to be long, preferentially affecting the ostial area of the coronary arteries, and the collagen and fibrin content in the formed inflammatory plaques are high and the fat content is low [62]. Acharya et al. in 2018 studied a case of coronary artery disease caused by radiotherapy for breast cancer and found severe, isolated bilateral coronary artery orifice lesions [66]. The typical manifestation of RICHD is that young patients with typical angina symptoms do not have risk factors for ischemic heart disease, and coronary artery occlusion involves more frequently the coronary artery opening and proximal coronary artery segment than the distal coronary artery segment [9, 48]. Although there are few traditional cardiovascular risk factors in patients with radiation coronary heart disease, if such risk factors such as hyperlipidemia exist, the progress of atherosclerosis might be accelerated [66]; and even though angina symptoms are common in patients with RICHD, nonanginal chest pain is the most common in the first 2–3 years after chest irradiation and/or mastectomy, accounting for approximately 50% of breast cancer survivors [9]. Patients with radiation coronary heart disease have a higher risk of asymptomatic myocardial infarction than the general population. The treatment of radiation-induced coronary artery disease, however, is no different. Cardiac surgery, including angioplasty, stenting, or bypass grafting may be used, but it may be more technically difficult and the procedure is more complex [67].

#### 1.4.3. Radiation-Induced Valvular Heart Disease

Early heart valve injury caused by radiation is usually manifested through the thickening and calcification of the valve and lobule. These abnormalities rarely show clinical symptoms, but in imaging studies, the detection rate of valvular lesions was 60% and it was as high as 80% in autopsies [67]. Radiation-induced valvular disease can develop from intimal thickening, no dysfunction to asymptomatic valvular dysfunction, and eventually symptomatic valvular stenosis or regurgitation [48]. Radiotherapy was found to increase the clinical risk of severe valvular disease, especially when the dose is above 30 Gy, with aortic valve involvement being the most frequent [68]. Aortic regurgitation is the most common valvular disease, but most patients show mild valve injury [13]. Aortic and mitral valves are more likely to be involved than tricuspid and pulmonary valves [8]. Valvular stenosis can occur 20 years after radiotherapy, and aortic stenosis is the most common type when valvular stenosis occurs [62]. Echocardiography has shown abnormal calcification of aortic intervalvular fibers and of the anterior mitral valve in patients with mild valve injury. Heart souffle and echocardiography suggest that valvular disease should be examined by echocardiography on a regular basis. Surgical treatment should be considered for patients with severe valvular disease [8].

#### 1.4.4. Radiation-Induced Conduction Dysfunction

Radiotherapy may affect the conduction system of the heart, leading to various types of arrhythmias [69]. The conduction system after radiation is rarely involved, but the range of the electrocardiogram after irradiation shows prolonged nonspecific ST-T, QT interval, decreased voltage, right bundle branch block, and sinus tachycardia [48]. Common conduction disorders occur quickly after radiation, including nonspecific repolarization changes, which are usually transient and asymptomatic, while severe abnormalities including atrioventricular node bradycardia, all levels of cardiac block, and sick sinus syndrome are known to occur many years after radiotherapy [48]. The abnormality of the conduction system may be caused directly by radiation or indirectly by the fibrosis and ischemia of the myocardium caused by radiation therapy, in which the right bundle branch block is most common because the right bundle branch is located in the vicinity of the endocardium and is most likely to be damaged during radiotherapy [48]. Complete heart block is a serious and delayed manifestation of radiation-induced cardiac damage, requiring pacemaker implantation [8, 13].

#### 1.4.5. Radiation-Induced Cardiomyopathy

Myocardial injury after radiation is related to microvascular injury, such as the decrease of microvessel density, the loss of alkaline phosphatase in vascular endothelial cells, and the increased expression of von Willebrand factor. Acute myocarditis is associated with inflammation caused by radiotherapy, accompanied by transient ST, T wave electrocardiogram abnormalities, and myocardial dysfunction [8]. Myocardial fibrosis induced by radiotherapy develops through the separation and replacement of proliferating collagen from the cardiomyocytes, which may lead to ischemic heart disease and even heart failure [2]. Myocardial fibrosis usually develops when a patient is exposed to high doses of radiotherapy, and radiation myocardial fibrosis is usually asymptomatic in the early stage and can be detected at least 10 years after radiotherapy [38]. In the late stage of myocardial injury, diffuse myocardial fibrosis complicated with severe diastolic dysfunction leads to restrictive cardiomyopathy [8]. Radiation-induced cardiomyopathy can be characterized by dilated or restricted phenotypes. But on the whole, radiotherapy-induced cardiomyopathy is more common than restrictive cardiomyopathy. The coexistence of radiation-induced cardiomyopathy and pericardial disease may be one of the causes of heart failure [13]. Cardiomyopathy is usually characterized by severe signs and symptoms, similar to constrictive pericarditis and severe heart failure. This serious sequela was evident in cases where no improvement after pericardiectomy was observed [67].

### 1.5. Prevention and Treatment of Radiation-Induced Cardiotoxicity

Cellular oxidative stress leads to the release of toxic free radicals from endothelial cells and vascular smooth muscle cells. These active species interact with cellular components, such as proteins, DNA, or lipids, leading to CVD. Therefore, cellular oxidative stress is the main pathogenic factor of CVD [14]. Oxidative stress reflects the imbalance between the oxidant and the antioxidant mechanism produced by the cells, and so the balance needs to be restored in order to reduce radiation-induced cardiac damage. Antioxidants are molecules that inhibit oxidative stress chain reactions and protect cell components from damage, as shown in Table 1. They are divided into enzymes and nonenzyme antioxidants. Enzyme antioxidants include SOD, GPX, glutathione reductase (GR), and CAT. Nonenzyme antioxidants based on their sources include exogenous and intrinsic antioxidants. GSH is an endogenous antioxidant; exogenous antioxidants include carotenoids, ascorbic acid (Vit C), *α*-tocopherol (Vit E), and flavonoids [14]. Antioxidants are the first line of defense against ROS damage. They can convert oxidants into less active substances and prevent lipids, proteins, and DNA in the cells from being damaged by ROS [25].

#### 1.5.1. Vitamin E

The longest known antioxidant in the biological system is *α*-tocopherol, which has a relatively high concentration in the cells and mitochondrial membranes [37]. *α*-Tocopherol is the most active form of vitamin E (Vit E) [15]. Various sources of food are believed to contain a large quantity of vitamin E, especially most of the nuts and seeds, such as almonds, peanuts, and sunflower seeds [70]. A large number of studies have shown that vitamin E, as a powerful antioxidant, has an important impact on cardiovascular events. Vitamin E can regulate the oxidative stress state. For example, *α*-tocopherol was reported to reduce the production of ROS by reducing the activity of NADPH oxidase, and Vit E interacted with ascorbic acid (Vit C) to terminate the free radical reaction [19]. Vit E is also an anti-inflammatory agent and its major anti-inflammatory effect is through the inhibition of NF-*κ*B. The anti-inflammatory effects of *α*-tocopherol include reducing the synthesis of ICAM-1 and VCAM-1, downregulating scavenging receptors, such as CD 36, reducing the release of IL-1B, and inhibiting the release of IL-1B in monocytes. Therefore, *α*-tocopherol can inhibit the adhesion of monocytes to endothelial cells and reduce their chances of entering the subendothelial space [19]. In addition, Vit E provides electrons to peroxide groups, which are produced in the process of lipid peroxidation. Therefore, Vit E can reduce the risk of coronary heart disease [14]. Many studies have identified the antioxidant and antiatherosclerotic properties of vitamin E in animals [71–74]. In 2001, Ferre et al. found that a diet containing vitamin E, palm oil, or sunflower oil would reduce the range of pathological changes in the aorta [71]. Some observational studies have also shown that a diet with higher vitamin E content is associated with a lower risk of coronary heart disease. In 1993, Rimm et al. found that men who took more than 60 IU of vitamin E per day had a significantly lower risk of coronary heart disease than men who consumed less than 7.5 IU per day [75]. In addition, men who took 100 IU vitamin E per day for at least 2 years had a 37% lower risk of coronary heart disease than men who did not take vitamin E, according to an observational study [76].

#### 1.5.2. Silymarin

Flavonoids comprise a wide range of polyphenolic antioxidants, which can be found in a variety of fruits and vegetables. Silymarin is one of the polyphenolic flavonoids extracted from milk thistle and belongs to nonenzyme antioxidants [77]. Various animal and human studies have shown that silymarin and its active components (silybin) exhibit strong antioxidant activities by scavenging free radicals, increasing glutathione concentration and SOD activity, and inhibiting lipid peroxidation [78, 79]. Silymarin, as an antioxidant, may prevent radiation-induced CVD in a variety of ways. For example, it inhibits ROS-producing enzymes in mitochondria and reduces the production of free radicals. Silymarin can also upregulate antioxidant enzymes and downregulate the activity of NADPH oxidase in the myocardium by stimulating the expression of Nrf2. Apart from the Nrf2 signal pathway, silymarin can also downregulate the NF-*κ*B pathway to reduce the production of proinflammatory cytokines and reduce the inflammatory response that stimulates atherosclerosis [14]. In 1999, Manna et al. showed that silymarin is an effective inhibitor of NF-*κ*B activation induced by a variety of inflammatory factors. Silymarin can inhibit NF-*κ*B activation by inhibiting the phosphorylation and degradation of IKB [79]. Silymarin, also as an herbal antioxidant, is used to prevent CVD, with fewer side effects, and can eliminate ROS and enhance antioxidant enzymes. In addition, Silymarin has a stronger antioxidant effect than vitamin E and other antioxidants [80].

#### 1.5.3. Resveratrol

Resveratrol is found mainly in grapes and red wine, as well as in plants and fruits, such as peanuts, cranberries, pistachios, blueberries, and blackberries. It can play a potentially protective role against CVD [81, 82]. Resveratrol is known for its antioxidative and anti-inflammatory properties. The mechanism through which resveratrol protects the heart is by increasing the activity of eNOS, which is beneficial to the generation of NO, the expansion of blood vessels, the reduction of platelet aggregation, and the inhibition of atherosclerosis [81]. In recent years, these characteristics have been widely studied in animal models and mannequin models, both in vivo and in vitro. As an antioxidant, resveratrol can improve the activity of the antioxidant enzyme and can be used as a free radical scavenger. Therefore, it can prevent DNA damage and cell membrane lipid peroxidation [82]. However, the direct scavenging effect of resveratrol on ROS is relatively poor, and the antioxidant properties of resveratrol in vivo are more attributed to its role as a redox gene regulator. It does not just reduce the uncoupling of NADPH oxidase and eNOS, but it also upregulates antioxidant defense enzymes [83]. Recent clinical trials have shown that resveratrol has low toxicity and good tolerance, and its pharmacological safety is as high as 5 g/day [84]. In 2012, Joao et al. found that dietary interventions supplemented with resveratrol significantly reduced many cardiac risk factors after 12 months at a low dose (8 mg/day) [85].

#### 1.5.4. Lycopene

Many red fruits and vegetables, including tomatoes, watermelons, pink grapefruit, apricots, pink guava, and papaya contain lycopene [29]. Lycopene is the most effective antioxidant among all kinds of common carotenoids. This carotenoid not only has free radical scavenging activity, but it also helps maintain the balance of the intracellular endogenous antioxidant defense system [25]. Lycopene was found to scavenge singlet oxygen, nitrogen dioxide, sulfur, and sulfonyl free radicals. In the process of singlet oxygen quenching, energy is transferred from singlet oxygen to the lycopene molecule and transformed into a triplet state rich in energy. The capture of other ROS leads to the oxidative decomposition of lycopene molecules. In this way, lycopene prevents the oxidation of lipids, proteins, and DNA in the body [25, 86]. In 2017, Gajowik and Dobrzyńska reported that the effect of adding lycopene one hour before irradiation to protect DNA from oxidative damage was higher than adding it immediately before radiation exposure. Moreover, it was found that the addition of lycopene after radiation could not reduce the damage level of DNA. However, lycopene supplementation, especially at low doses, can be used to protect DNA from radiation-induced oxidative damage [86]. A study by Gunasekaran et al. in 2013 also showed that the addition of lycopene can reduce the level of oxidative stress by inhibiting the formation of ROS and the oxidation of glutathione [87]. Lycopene was also found to exhibit an anticancer activity by reducing cell proliferation induced by insulin-like growth factor in various cancer cells [25]. Therefore, lycopene seems to be a promising natural antioxidant candidate for radiation protection and cancer prevention in normal cells [88].

#### 1.5.5. Melatonin

Melatonin is a natural hormone found in plants, such as cereals, olives, walnuts, tomatoes, pineapples, ginger, and beans. Melatonin has a strong radiation protection effect because of its strong antioxidant ability [63]. Different animal studies of melatonin in various physiologic and pharmacologic concentrations and representing a wide range of doses have shown that melatonin has very low toxicity [89]. Melatonin may have protective effects on the heart through direct free radical scavenging and indirectly through its antioxidant properties [90]. The mechanism of melatonin scavenging free radicals seems to be different from most of the other antioxidants because melatonin interacts with ROS without stimulating the redox system; furthermore, melatonin converts free radicals into stable products, which basically do not react with other molecules [26]. In addition, the antioxidant effect of melatonin involves the donation of two electrons, which do not become free radicals after providing electrons to free radicals and neutralizing free radicals to protect cells from free radical attacks. In other words, melatonin is different from other antioxidants such as vitamin E, because the reaction product of melatonin with free radicals is an antioxidant in itself [91]. The indirect effect of melatonin is to stimulate antioxidant defense, including GSH, GPX, SOD, GR, and CAT [92]. Several studies have shown that melatonin can enhance the activity of glutathione peroxidase and increase the synthesis of glutathione [93]. Melatonin can also inhibit the translocation of NF-*κ*B into the nucleus, thus inhibiting the signal pathway mediated by NF-*κ*B and protecting the integrity of mitochondria by shielding them from radiation-induced DNA damage, so as to reduce the oxidative stress induced by ROS after radiation [26]. In 2014, Iclal et al. studied the effect of melatonin on the prevention of radiation heart disease in rats by intraperitoneally injecting 50 mg/kg melatonin for 15 min before exposure to a single dose of 18 Gy. Compared to the untreated group, the histopathological evaluation at 6 months after irradiation showed that melatonin prevented vasculitis and lowered muscle cell necrosis and fibrosis [94]. In 2019, Farhood et al. reported that IL-4 and its downstream genes, *IL4ra1*, *DUOX1*, and *DUOX2* were activated after rat heart irradiation. Melatonin was found to inhibit the increased expression of *IL-4*, *IL4ra1*, *DUOX1*, and *DUOX2* before irradiation and may have protective effects on heart injury induced by ionizing radiation by inhibiting the expression of *IL-4*, *DUOX1*, and *DUOX2* [63].

#### 1.5.6. Hesperidin

Hesperidin is a bioflavonoid found in citrus fruits, such as oranges, lemons, and grapes, as well as in plant extracts, such as tea and olive oil. The pericarp of these plants has the highest concentration of hesperidin. Hesperidin, as a natural antioxidant, has been reported to reduce radiation-induced heart damage [30]. The level of oxidative stress in the heart of mice fed with hesperidin was significantly decreased, such as the increased expression of antioxidant enzymes, including SOD1 and SOD2, and passivation of NADPH oxidase. More importantly, hesperidin plays an important role in improving cardiac hypertrophy and cardiac remodeling by inhibiting TGF-*β*1 mRNA expression and Smad2/Smad3 phosphorylation [95]. In 2016, Rezaeyan et al. found that the level of oxidative damage and inflammation were decreased by oral administration of 100 mg/kg hesperidin for 7 days before a single dose of *γ*-ray irradiation with 18 Gy in the chest of rats [96]. Compared with irradiated mice without hesperidin treatment, treatment with hesperidin in mice for 7 days after radiotherapy significantly decreased the level of protein carbonyl groups and improved the levels of lipid peroxide and glutathione in the heart, which were induced by radiation in a dose-dependent manner [97]. In addition, it was found that hesperidin could protect the heart of aged rats by mediating the expression of Nrf2, upregulating the activity of antioxidant enzymes and reducing the macromolecular damage induced by oxidative stress [98].

## 2. Limitations and New Research Progress on Antioxidants

Endogenous antioxidants play a crucial role in maintaining optimal cellular function. However, under oxidative stress conditions, endogenous antioxidants may not be sufficient and the addition of dietary antioxidants may be required. Antioxidant activity depends on some special conditions, particularly the dose in cells and redox conditions of antioxidants [39]; high doses of antioxidants may alter redox balance and lead to cellular dysfunction by interacting with ROS at physiological concentration [99]. In the following article, we take vitamin E, melatonin, and resveratrol as examples. In terms of biological effects, the most active form of vitamin E is *α*-tocopherol, which is also the most abundant form in nature and has a strong antioxidant effect. Nevertheless, recent in vitro studies have shown that vitamin E may promote oxidation at a high dose. A meta-analysis suggests that human adults with chronic diseases who consume more than 400 U a day of vitamin E supplements increase all-cause mortality [100]. In addition, melatonin has a strong radiation protection effect owing to its strong antioxidant ability. However, studies have shown that a high concentration of melatonin induces intracellular ROS production and accumulation, exhibiting a prooxidant effect that plays an upstream role in mitochondria-mediated apoptosis and autophagy. The results indicate that melatonin at high concentration was a potential adjuvant in patients with head and neck squamous cell carcinoma who were undergoing radiotherapy [101]. Resveratrol is known for its antioxidant properties, but it has been identified as a cytotoxic and prooxidant compound, depending on its concentration, exposure time, and cell type [102]. Although the dose-dependent oxidative effect of resveratrol leads to oxidative stress of cells treated with 50 ml, the lower cytotoxicity found at 120 h of treatment may indicate that the surviving cells seem to be more resistant to resveratrol-induced oxidative damage, which seems to weaken with the prolongation of treatment time [103]. To sum up, it is generally accepted that each antioxidant is actually a redox, and therefore, may become a prooxidant, accelerating lipid peroxidation and/or inducing DNA damage under specific conditions. Therefore, it is strongly suggested that this prooxidant effect may be an important mechanism in the anticancer effect of resveratrol and induction of apoptosis [102].

## 3. Conclusions

Accumulating clinical evidence suggests that exposure to ionizing radiation increases the risk of CVD. Oxidative stress plays an important role in radiation-induced cardiac toxicity. Oxidative stress is the result of excessive production of ROS and the decrease of ROS scavenging ability. It promotes oxidative damage of DNA, which is characterized by cardiac function damage. Cancer survivors who initially received radiotherapy are at increased risk of multiple manifestations of CVD. Although modern radiotherapy has adopted cardiac protection techniques that are likely to reduce the prevalence and severity of CV complications, more efforts are needed to prevent and treat these side effects. Therefore, in order to improve the survival rate and prognosis of cancer patients treated with radiotherapy, the use of natural antioxidants to eliminate excessive ROS, inhibit the production of ROS, and inhibit oxidative stress is a promising strategy for the treatment of radiation-induced heart disease.

## Figures and Tables

**Table 1:** Antioxidants and their effects.

Antioxidant	Source	Mechanism	Effect
Vitamin E	Amygdala, peanut, and sunflower seeds [70]	Reduce the activity of NADPH oxidase and inhibit lipid peroxidation and downregulate NF-*κ*B [19]	Antioxidation, anti-inflammation, and a decline of the risk of coronary heart diseases [75, 76]
Silymarin	Milk thistle [77]	Stimulate the expression of Nrf2 and downregulate NF-*κ*B [14]	Suppression of oxidative stress and inflammation and antiatherosclerosis [14, 80]
Resveratrol	Grapes and red wine [81, 82]	Reduce the uncoupling of NADPH oxidase and eNOS and upregulate antioxidant defense enzymes [81, 82]	Antioxidative and anti-inflammatory properties against cardiovascular diseases [81, 82, 85]
Lycopene	Tomatoes, watermelons, pink grapefruit, apricots, pink guava, and papaya [29]	Scavenge singlet oxygen, nitrogen dioxide, sulfur, and sulfonyl-free radicals [25]	Prevention of the oxidation of DNA, lipid, and proteins [25, 86]
Melatonin	Cereals, olives, walnuts, tomatoes, pineapples, ginger, and beans [63]	Donate two electrons directly and stimulate the enzymatic antioxidant system indirectly [26, 91]	Suppression of oxidative stress and prevention of RIHD [90, 91]
Hesperidin	Oranges, lemons, grapes, and olive oil [30]	Passivate NADPH oxidase and inhibit TGF-***β***1 mRNA expression [95]	Inhibition of oxidative stress, cardiac hypertrophy, and cardiac remodeling [95, 96]
